# Wharton’s Jelly-derived Mesenchymal Stem Cells can Differentiate into Hepatocyte-like Cells by HepG2 Cell Line Extract

**Published:** 2015-03

**Authors:** Maryam Borhani-Haghighi, Tahereh Talaei-Khozani, Maryam Ayatollahi, Zahra Vojdani

**Affiliations:** 1 Laboratory for Stem Cell Research, Department of Anatomy, School of Medicine, Shiraz University of Medical Sciences, Shiraz, Iran;; 2 Transplantation Research Center, Shiraz University of Medical Sciences, Shiraz, Iran

**Keywords:** Wharton’s jelly, Mesenchymal stem cells, Cell differentiation, Cell-free system

## Abstract

**Background:**

Wharton’s jelly is an unlimited source of stem cells that can be used in cell therapy and tissue engineering without any ethical concern. It has been revealed the cell-free extract could be effective to induce cell differentiation. The objective of this study was to induce Wharton’s jelly-derived mesenchymal stem cells (MSCs) into hepatocyte-like cells by premeabilization of the cells in the presence of HepG2 cell line extract.

**Methods:**

MSCs were isolated from the umbilical cord, CD marker profile and their differentiation potential into adipogenic and osteogenic lineages were determined. The cells were then, permeabilized by streptolysin O in the presence of HepG cell extract. The treated cells were cultured for 17 days. The cell phenotype was evaluated and the hepatocyte specific markers were detected by immunofluorescence and immunocytochemistry. The Periodic Acid Schiff (PAS) reaction and the cellular uptake of indocyanine green were performed to evaluate the functional behavior of the differentiated cells.

**Results:**

The phenotype of extract-treated MSCs changed into a round or polygonal cells with few short processes and they could express high level of albumin, cytokeratin 18 and 19. The MSCs also could store glycogen and uptake and release indocyanine green.

**Conclusion:**

We demonstrated for the first time that Wharton’s jelly-derived MSCs could differentiate into hepatocyte-like cells by premeabilization of them in the presence of HepG2 cell extract. This study suggests a feasible method to differentiate MSCs into functional hepatocyte-like cells.

## Introduction

Whole or partial liver transplantation is the only effective treatment for many hepatic diseases. Organ transplantation can be replaced by cell therapy. The shortage of the appropriate donor encourages researchers to find new sources for cell therapy. Hepatocyte differentiation from mesenchymal stem cell (MSC) can replace organ transplantation. Hepatocytes can be differentiated by supplementation of the culture media with a combination of growth factors,^1^^,^^2^ small molecules,^1^ or chromatin modifying agents.^2^

Wharton’s jelly-derived MSCs as medical waste after delivery, is a rich source of stem cells and can be used in regenerative medicine without any ethical concern. Stable karyotype,^3^ the highest expansion potential among various MSCs,^4^ their immunomodulatory potential^5^ and lack of tumorigenesity^6^ make the Wharton’s jelly-derived MSCs as an attractive source for transplantation. It has been demonstrated that MSC isolated from Wharton’s jelly could express both MSC and embryonic stem cell (ESC) markers.^7^ Wharton’s jelly-derived MSCs can differentiate to all three germ lineages^8^ and also express the markers of endoderm along with mesoderm and ectoderm.^9^ Naive Wharton’s jelly-derived MSCs have been shown to express a low level of some hepatocyte markers. The MSCs from umbilical cord has been detected to be able to differentiate toward low immunogenic and functional hepatocytes in vivo^10^ and in vitro.^11^^,^^12^ With regard to these considerations, it seems that Wharton’s jelly-derived MSCs can be an appropriate source of stem cell for liver replacement therapy.

Liver specification begins with binding the endoderm specific transcription factors such as GATA4, to the enhancer of the early liver specific genes.^13^ Transcription factors such as HNF4 regulate the expression of serum factors and metabolic enzymes secreted from hepatocye.^14^ Wharton’s jelly-derived MSCs express some early liver specific markers; therefore, they could differentiate into the functional hepatocytes more feasible than stem cells from the other sources. Cell-free extract from HepG2 cell line contains nearly all transcription factors necessary for induction of a cell type toward hepatogenic lineage. Differentiation or transdifferentiation can also be mediated by temporal permeabilization of the cells in the presence of tissue extracts by streptolysin O or lipofection. Transdifferentiation of mouse fibroblast^15^^,^^16^ and human granulose cells^17^ into induced pluripotent stem cells, human lymphocyte^18^ and MSCs^19^ into cardiomyocytes and HepG2 cell line into insulin-producing cells^19^ were performed by permeabilization of the cells in the presence of cell-free extract. The stem cells from Infrapatellar fat pad of patients with osteoarthritis^20^ and bone marrow^21^ were also permeabilized in the presence of chondrocyte extract and were induced to differentiate to chondrocyte. This study was conducted to find whether the content of the cell-free extract from hepatocyte cell line, HepG2, could induce the MSCs isolated from Wharton’s jelly toward functional hepatocytes.

## Materials and Methods

This study was an experimental interventional study. Umbilical cords from healthy infants were transferred to the laboratory within 4-24h after delivery via cesarean section with informed consents from the infants’ parents. The specimens were prepared from Hafez and Shafa hospitals (Shiraz, Iran) between 2011-2013. The experimental design was in accordance with the guidelines of the Ethics Committee of Shiraz University of Medical Sciences. The umbilical cords were washed with phosphate buffer saline (PBS) containing 5% penicillin/streptomycin. A longitudinal section was made through the umbilical vein and the endothelial cells were scratched and discarded. The umbilical arteries were removed and the rest was cut into 0.5-1 cm pieces. Each piece was put into a 100 mm petri dish and cultured in the presence of α-minimum essential medium (α-MEM) containing 10% fetal calf serum (FCS), 0.1% L-glutamine and 0.1% penicillin /streptomycin for 8-10 days. Upon confluency, the cells were passaged.

*Cell Characterization*

The harvested cells were washed in PBS and incubated in blocking buffer containing10% goat serum. The cells were exposed to FITC-conjugated anti-CD44, CD144, PE-conjugated anti-CD106, CD34 and preCP conjugated anti-CD105 antibodies (all from Abcam, Cambridge, UK). The cells were then fixed in 4% paraformaldehyde. The appropriate isotype control antibodies were used to exclude background staining. The percent of stained cells were evaluated by a four-color FACSCalibur flow cytometry machine. The graphs were depicted by WinMed software.

Assessment of Differentiation Potential

To evaluate the osteogenic and adipogenic potential of the Wharton’s jelly-derived MSCs, the cells were exposed to the NH OsteoDiff Medium (MACS, Germany) and human adipogenic stimulatory supplements (StemCell Technologies Inc, Canada) for 4 weeks. The cells were then stained with alizarin red S and oil red to detect the calcium deposition and adipocyte formation, respectively. 

*Cell-free Extract Preparation*

HepG2 cell line was purchased from Pasteur institute, Iran. The cells were grown in the presence of Roswell Park Memorial Institute medium (RPMI) containing 10% FCS, 0.1% L-glutamine and 0.1% penicillin-streptomycin. The harvested cells were washed in PBS and then in Hank’s buffered salt solution (HBSS). The cells were then incubated in cold lysis buffer (containing 50 mM NaCl, 5 mM MgCl_2_, 20 mM HEPES, 1 mM Dithiothreitol and protease inhibitor (Sigma), pH 8.2) for 45 min. The cells were then sonicated by pulse sonicator (Hielscher). The lysate was centrifuged at 15000 g for 15 min at 4°C. The supernatant was checked for lack of the whole cell existence. The supernatant was aliquoted and snap frozen in liquid nitrogen. The extract was stored at -80°C until used.

The protein yield of the extract was evaluated by Bradford protein assay. The protein concentration in the extract was calculated as 283 mg/mL.

*Permeabilization Assay*

Aliquot of cell suspension containing 20000 cells were permeabilized in the presence of 920 ng/mL of streptolysin O (Sigma) and FITC-conjugated 70,000 Mr dextran (Sigma) in HBSS for 50 min at 37°C. The uptake of FITC-conjugated dextran was detected by fluorescent microscopy.

*Cytotoxicity Assay*

Twenty µL of the serial dilution of the extract containing 50,000 viable cells were incubated for 1 h at 37°C. Trypan blue exclusion technique was used to determine the number of viable and dead cells. For control culture, the same number of viable cells were exposed to the solvent of the extract, HBSS, and incubated at the same condition. Neutral red assay was performed to detect the cell viability. The viable cells were stained red due to the dye uptake by lysosome.

*Cell Permeabilization in the Presence of Extract*

The harvested MSCs were washed with cold PBS without Ca^2+^ and Mg^2+^(PBS^-^) twice and resuspended in HBSS. An aliquot of 20,000 cells per 16.4 µL of cold HBSS incubated at 37°C for 2 min and then 4.6 µL of streptolysin O was added and incubated for 50 min at 37°C. The final concentration of streptolysin O was 920 ng/mL. Then, 20 µL of extract containing ATP regeneration system (an equal volume of ATP, GTP, creatine phosphate and creatine kinase (all from sigma) and 25 mM NTPs) were added to the cells and incubated for additional 1h at 37°C.

The cells were then exposed to culture medium containing 2 mM CaCl_2 _and cultured in a 24 well culture plate. The permeabilized cells were cultured for 17 days. The control cultures treated with the extract-free HBSS containing streptolysin O and ATP regeneration system. They were also incubated in the same condition with extract-treated cultures.

*Immunocytochemistry*

Immunoperoxidase method was used to detect cytokeratin 18 and 19. The cells were fixed with 10% paraformaldehyde for 20 min. Endogenous peroxidase were blocked by 0.3% H_2_O_2_ in distilled water for 20 min. The non-specific binding sites were blocked by treating the cells with PBS containing 10% gout serum for 20 min and then, incubated in mouse anti-human cytokeratin 18 (ready to use) and 19 (at a dilution of 1:20) (both from DAKO) for 45 min. The samples were then treated with super enhancer reagent (HK518-50K, DAKO, BioGenex, Fremont, CA, USA) for 15 minutes at room temperature. The cultures were incubated with dextran polymer-horseradish peroxidase (HRP) (BioGenex) for 30 min at room temperature. 3,3′-Diaminobenzidine (DAB) was used as chromogen (DAKO). The cultures were counterstained with hematoxylin for 1 min.

The specimens were also stained for albumin by immunofluorescence. The cells were fixed with 4% paraformaldehyde and background staining were blocked with PBS containing 4% gout serum, 5% bovine serum albumin (BSA) and 1% Tween-20 for 20 min. The cells were then incubated in FITC-conjugated anti-albumin antibody (DAKO, Denmark) at a dilution of 1:30 for 1 h at room temperature. After washing, the cells counterstained with 4’,6-diamidino-2-phenylindole (DAPI) and mounted in PBS. 

*Periodic Acid–Schiff Staining (PAS)*

Glycogen storage of hepatocyte-like cells was evaluated by PAS staining. The experiment and control cultures were fixed with 10% paraformaldehyde. After washing, the specimens were incubated in 0.5% periodic acid for 5 min. The glycogen content of the cells was then visualized by incubating the cells in Schiff’s reagent. The cells were then counterstained with hematoxylin. 

*Indocyanine Green*

The culture media were replaced with 1 mg/mL of indocyanine green prepared in culture media. The cultures were incubated at 37°C for 15 min. The media containing indocyanine green was then discarded and micrographs were taken from the cultures. The cells were incubated for 6 h in fresh medium and the releasing of the indocyanine green was evaluated. HepG2 cell line was considered as positive control. 

*Statistical Analysis*

The data were analyzed by Mann-Whitney test and a P-value less than 0.001was considered as significant difference. The data were analyzed by SPSS 16.0 for windows. All experiments were performed in triplicate. 

## Results

*Phenotype Analyses*

**Figure 1:**
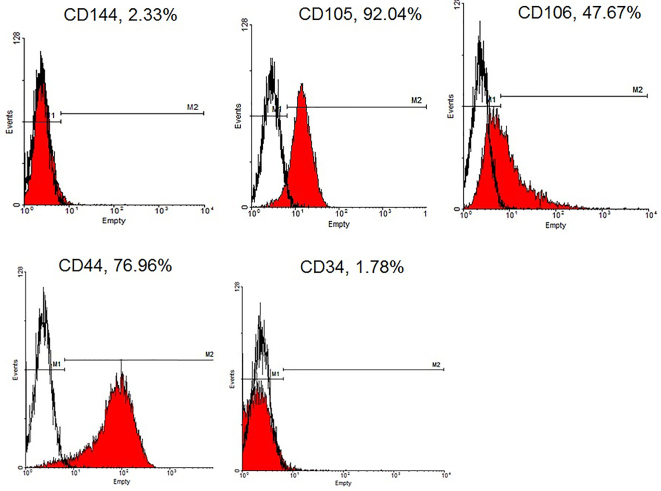
The flow cytometry showed the Wharton’s jelly derived-MSCs could express CD106, CD 44 and Cd105; however, the frequency of the cells expressed CD44 and CD34 was negligible.

**Figure 2:**
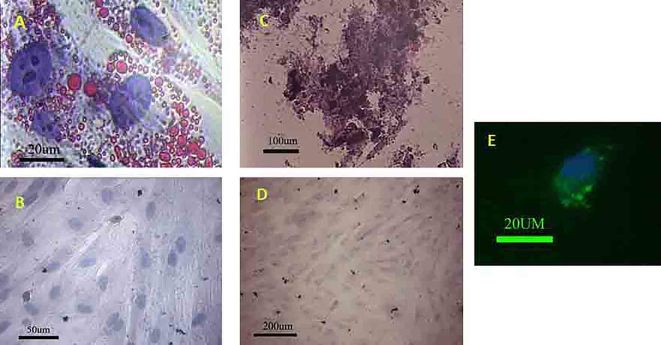
(A) The MSCs could differentiate into adipocyte after exposing to the adipogenic media. (B) Negative control did not show any lipid droplet; oil red staining. The MSCs showed the potential to differentiate toward osteogenic lineage. (C) The red part contains calcium that reacted with alizarin red S. (D) Negative control did not show any Ca deposition; alizarin red S staining. MSCs premeabilized in the presence of FITC-dextran. (E) The green granule indicated the FITC-dextran was internalized by MSC.

Wharton’s jelly-derived MSCs could express CD44, CD105 and CD106 (76.96%, 92.4% and 47.67%; respectively); however, the percentages of the cells expressed CD144 and CD34 were negligible (figure 1). The histochemical staining was also revealed the multipotential capability of the MSCs. They could differentiate toward osteoblasts and adipocytes in the presence of osteogenic and adipogenic media, respectively (figure 2A-D).

*Permeabilization Assay*

The permeabilization of the cells was evaluated by treating the cells with FITC-conjugated dextran with the same range of molecular weight with protein content of the extract in the presence of streptolysin O. FITC-conjugated dextran internalization was shown in the permeabilized cells (figure 2E).

*Morphological Observations*

Microscopic observations showed the cell morphology modified in extract-treated cultures. Some round small cells or cells with few processes along with fibroblast-like cells were appeared in the culture plate. The cell size was smaller after being treated with extract compared with non-treated cells. The cell phenotype was almost similar to HepG2 cells morphology. 

*Hepatocyte Markers*

**Figure 3:**
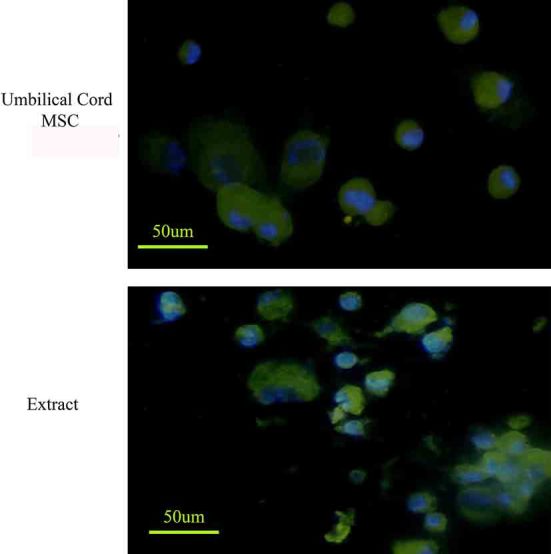
The Wharton’s jelly MSCs expressed albumin; however, after permeabilization the cells in the presence of extract, the cells reacted with anti-albumin antibody more intense.

**Figure 4:**
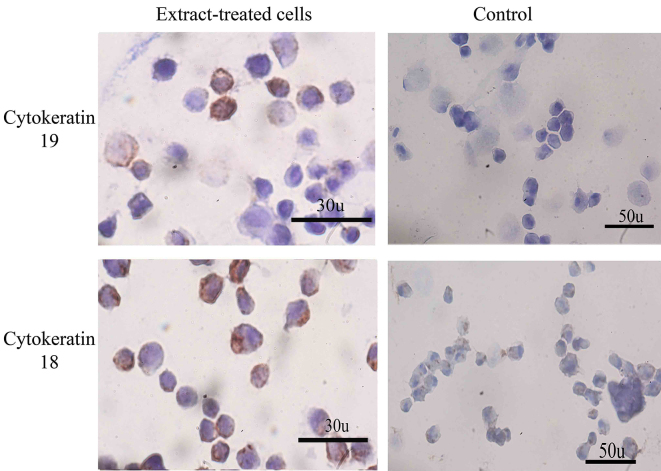
The extract-treated cells were stained more intensely with anti-cytokeratin 19 and 18. However, the control cells also express a low level of these two hepatocyte markers, the expression level was higher in extract-treated cells.

Immunostaining showed both extract-treated and control cells could express albumin; however, the intensity of the reaction of anti-albumin antibody was “stronger” in extract-treated than control cultures (figure 3). The extract-treated cells could also express cytokeratin 18 and 19 (figure 4). The frequency of the extract-treated cells expressed cytokeratin 18 and 19 were 76.66±7.76 and 52.33±4.16; respectively. The control cultures also reacted for cytokeratin 18 and 19 and the percentages of positive cells were 25.33±4.04 and 10.33±2.08; respectively. The statistical analysis showed a significant increase in the percentages of cytokeratin 18- and 19-positive cells compared with those in control cultures (P<0.001).

**Figure 5:**
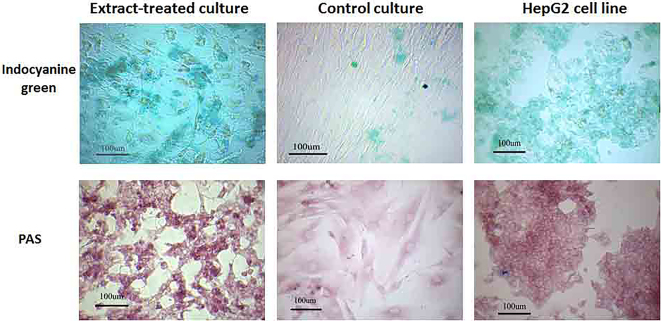
Some extract-treated cells could uptake indocyanine green and store glycogen. The round cells with few short processes showed phonotype similar to HepG2 cells and could uptake indocyanine green and PAS-positive.

PAS staining showed very low intense of PAS-positive reacted cells in control cultures; however, extract-treated cells could store more glycogen; and therefore, they reacted “strongly” with PAS assay. The treated cells also could uptake and release the indocyanine green (figure 5). These tests showed the different factors in the extract could induce the MSCs differentiate into functional hepatocyte-like cells. Non-treated cells could not uptake indocyanine green.

## Discussion

The capability of the Wharton’s jelly-derived MSCs toward hepatocytes was demonstrated previously by other researchers.^11^^,^^12^ The finding of this study along with the others has been demonstrated that umbilical cord MSCs could express a low level of some hepatocyte markers.^10^^-^^12^ It has been reported that Wharton’s jelly-derived MSCs could not express adult hepatic markers; however, the constitutive expression of transcription factors involved in liver development along with liver progenitor markers were detected in this type of MSCs.^22^ In vitro and in vivo studies suggested various protocols for differentiating these stem cells into hepatocyte-like cells and the functional tests showed that they could be also functional.^23^^,^^24^ Immonomodulatory features, high proliferation capacity^25^ and hepatocyte marker expression^10^ made Wharton’s jelly MSCs an appropriate candidate for liver cell therapy.

Cell differentiation can be induced by various techniques. Treating the cells with culture media containing appropriate growth factors induced the MSCs derived from umbilical cord toward functional hepatocyte-like cells.^23^^,^^26^ A combination of hepatocyte growth factor, oncostatin M and dexamethasone with the other growth factors and cytokines such as insulin-like growth factor,^4^ fibroblast growth factor-4^27^ in bone marrow-derived MSCs and also with basic fibroblast growth factor,^28^ DMSO^29^ in adipose-derived MSCs. Besides, oncostatin M has been reported to replace by leukemia inhibitory factor for differentiating bone marrow-derived MSCs toward hepatocyte lineage.^30^ However, there are a few number of researches focusing on Wharton’s jelly MSCs differentiation toward hepatocyte; but it has been shown that they could differentiate into hepatocyte by treating with HGF, OSM along with FGF-4^23^ or bFGF.^11^

Supplementation of the culture media with cell-free extract has been demonstrated to induce cell proliferation and changed cell differentiation capacity toward hepatocyte.^31^ It was also shown the tissue extract from damaged liver could induce rat bone marrow-derived MSCs to hepatocytes.^32^ The extract from damaged liver contains many growth factors necessary for hepatocyte differentiation. However, it is not clear whether these differentiated cells are functional.^33^ Cell-free extract also contains transcription factors necessary for expression of adult tissue specific markers. Some researchers permeabilized the cells in the presence of cell-free extract to induce the dedifferentiation or transdifferentiation of the adult somatic cells toward various cell lineages such as cardiomyocytes,^18^ chondrocytes,^20^^,^^21^ insulin-expressing cells^34^ or induced pluripotent stem cells.^17^ Our data also showed MSCs could differentiate toward functional hepatocyte-like cells when they permeabilized in the presence of HepG2 cell line extract. The cells showed a morphological phenotype similar to HepG2 cell line after permeabilization and could store glycogen and uptake and release indocyanine green.

Permeabilization of the cells in the presence of cell-free extract led to an increase in the frequency of the cells expressed the hepatocyte markers compared to non-treated cultures. The cells were also stained more intense compared with non-treated cells. The functional evaluation of the extract-treated cells revealed a positive staining for PAS and indocyanine green assays. Extract from HepG2 cell line was detected to enhance protein synthesizing activity and induce endogenous mRNA transcription.^35^ Hepatic nuclear factor 4 (HNF4) is one of the important transcription factor that targets the liver specific genes and present in liver extract.^36^ Such a transcription factor may be responsible for the enhancement of hepatocyte marker expression after permeabilization of the cells in the extract-treated cultures.

## Conclusion

This study has shown a simple and cheap method for generating functional hepatocyte-like cells from Wharton’s jelly-derived MSCs. The expression of a high level of hepatocyte markers and the ability to store glycogen and uptake indocyanine green revealed cell differentiation achievement. Wharton’s jelly-derived MSCs could differentiate into functional hepatocytes easily. The MSCs from umbilical cord expressed endodermal and hepatocyte progenitor markers; and therefore, can be considered as an appropriate cell source for tissue engineering and cell therapy. 
